# More in hope than expectation: a systematic review of women's expectations and experience of pain relief in labour

**DOI:** 10.1186/1741-7015-6-7

**Published:** 2008-03-14

**Authors:** Joanne E Lally, Madeleine J Murtagh, Sheila Macphail, Richard Thomson

**Affiliations:** 1 Institute of Health and Society, The Medical School, Newcastle University, Newcastle upon Tyne NE2 4HH, UK; 2 Women's Services, 3rd Floor Leazes Wing, Royal Victoria Infirmary, Newcastle upon Tyne NE1 4LP, UK

## Abstract

**Background:**

Childbirth is one of the most painful events that a woman is likely to experience, the multi-dimensional aspect and intensity of which far exceeds disease conditions. A woman's lack of knowledge about the risks and benefits of the various methods of pain relief can heighten anxiety. Women are increasingly expected, and are expecting, to participate in decisions about their healthcare. Involvement should allow women to make better-informed decisions; the National Institute for Clinical Excellence has stated that we need effective ways of supporting pregnant women in making informed decisions during labour. Our aim was to systematically review the empirical literature on women's expectations and experiences of pain and pain relief during labour, as well as their involvement in the decision-making process.

**Methods:**

A systematic review was conducted using the following databases: Medical Literature Analysis and Retrieval System Online (MEDLINE), Cumulative Index to Nursing and Allied Health Literature (CINAHL), Bath Information and Database Service (BIDS), Excerpta Medica Database Guide (EMBASE), Midwives Information and Resource (MIDIRS), Sociological Abstracts and PsychINFO. Studies that examined experience and expectations of pain, and its relief in labour, were appraised and the findings were integrated into a systematic review.

**Results:**

Appraisal revealed four key themes: the level and type of pain, pain relief, involvement in decision-making and control. Studies predominantly showed that women underestimated the pain they would experience. Women may hope for a labour free of pain relief, but many found that they needed or benefited from it. There is a distinction between women's desire for a drug-free labour and the expectation that they may need some sort of pain relief. Inaccurate or unrealistic expectations about pain may mean that women are not prepared appropriately for labour. Many women acknowledged that they wanted to participate in decision-making, but the degree of involvement varied. Women expected to take control in labour in a number of ways, but their degree of reported control was less than hoped for.

**Conclusion:**

Women may have ideal hopes of what they would like to happen with respect to pain relief, control and engagement in decision-making, but experience is often very different from expectations. Antenatal educators need to ensure that pregnant women are appropriately prepared for what might actually happen to limit this expectation-experience gap and potentially support greater satisfaction with labour.

## Background

Childbirth is one of the most painful events that a woman is likely to experience, the multi-dimensional aspect and intensity of which far exceeds disease conditions [1,2]. It is therefore not surprising that many pregnant women have concerns about the pain they will encounter and the methods of pain relief that are available during labour. Women's lack of appropriate knowledge about the risks and benefits of the various methods of pain relief can heighten anxiety [3,4].

Women are increasingly expected, and are expecting, to participate in decisions about their healthcare, including in pregnancy and childbirth [5-7]. There are choices to be made during pregnancy about options available for pain relief in labour; each method has its own risks and benefits, with variations in effectiveness, availability and acceptability. Wennberg and others have argued that unexplained variations in practice in the face of uncertainty should lead to greater involvement of patients in decision-making. They argued that this involvement should allow patients to make better-informed decisions by presenting both the clinical evidence and the likely effects of alternative interventions [8-10]. These recommendations, however, may not be appropriate or indeed feasible for women during the actual process of labour.

One way of supporting patients in the decision-making process has been the introduction of patient decision aids [11,12]. A systematic review of evaluations of decision aids concluded that they improve knowledge, reduce decisional conflict and engage patients more actively in decision-making, but have little effect on satisfaction and a variable effect on the actual decisions made [13]. Although a great deal of information is made available to women throughout their pregnancy, and there are several published Cochrane reviews on the effectiveness of specific interventions, [14-16] there is limited use of decision aids to assist women when making decisions regarding pain relief in labour [17,18]. Recent guidelines on routine care for the health of pregnant women, published by the National Institute for Clinical Excellence (NICE), suggest that there is an urgent need to fill a gap in knowledge by undertaking research on effective ways of helping health professionals to support pregnant women in making informed decisions during labour [19,20], also that healthcare professionals should consider how their own values and beliefs inform their attitude to coping with pain in labour and ensure their care supports the woman's choice [21].

A systematic review has been published on women's satisfaction with the experience of childbirth which provides some insight into women's expectations and experience of pregnancy [22]. It identifies four key factors which influence satisfaction: personal expectations, the amount of support from caregivers, the quality of the caregiver-patient relationship and involvement in decision-making; for example, an increase in involvement in decision-making led to a greater degree of satisfaction. These factors appear to be so important that they override the influences of age, socioeconomic status, ethnicity, childbirth preparation, the physical birth environment, pain, immobility, medical interventions and continuity of care when women evaluate their childbirth experiences [22].

When an initial literature search on pain relief in labour was undertaken, it was apparent that there was a discrepancy between women's expectations of pain and of methods of pain relief and their actual experience. There also appeared to be a similar mismatch between women's expectations and their actual involvement in decision-making. As a result, this systematic review was undertaken in order to address the following questions. What are women's expectations about pain, its relief during labour and their involvement in the decision-making process? Are expectations met by women's experiences? To date no systematic review has been conducted on these questions.

## Methods

Combinations of key words used in this literature search were childbirth, labour (labor), pain, pain relief, obstetric analgesia, experience and expectations. Studies of both pharmacological and non-pharmacological methods of pain relief were considered. The following literature databases were searched using these key words: Medical Literature Analysis and Retrieval System Online (MEDLINE, 1966–2007), Cumulative Index to Nursing and Allied Health Literature (CINAHL, 1982–2007), Bath Information and Database Service (BIDS, 1951–2007), Excerpta Medica Database Guide (EMBASE, 1980–2007), Midwives Information and Resource (MIDIRS), Sociological Abstracts (1963–2007) and PsychINFO Medline (1906–2007). The Cochrane database of systematic reviews and grey literature was also searched. Publications were limited to the English language only. Searches were performed of the references of the key papers included in the review.

The review identified studies using both qualitative and quantitative methods; both have been included in this review in order to provide a comprehensive integrative overview of the current evidence. Studies were included if they used recognised robust methods to investigate or describe women's experiences and/or expectations about pain relief and the decision-making process. Studies were excluded if the focus was on a specific type of pain, a measurement of pain or another aspect of labour. Personal accounts and theoretical papers about childbirth were also excluded. It should be noted that expectations, experiences and decision-making in the quantitative papers were often a secondary outcome; papers where this was the case were also included as they were still able to provide important information that was relevant to the review question. All qualitative papers were assessed in terms of validity, methods used and analysis of the results, using the Critical Appraisal Skills Programme (CASP) appraisal tool for qualitative research [23] (Table 1). For quantitative papers a framework for appraising a survey [24] was adapted for the needs of this review (Table 2).

**Table 1:** Appraisal tool for qualitative papers

Was the aim or the research question clear?
Is a qualitative methodology appropriate?
*Detailed questions*
Was the research design appropriate to address the aims of the research?
Was the recruitment strategy appropriate to the aims of the research?
Were the data collected in a way that addressed the research issue?
Is there evidence of reflexivity?
Have ethical issues been taken into account/have the ethical implications been considered appropriately?
Was the data analysis sufficiently rigorous?
Is there a clear statement of findings?
How valuable do we think the research is to this body of knowledge?

**Table 2:** Appraisal tool for quantitative papers

What was the response rate?
What question is the study aiming to answer?
Was the survey specifically designed with this question in mind?
Does the survey measures used allow this question to be answered clearly?
Is the population surveyed described clearly?
How was the survey carried out?
Is the denominator reported?
Are the measures reported objective and reliable?
Are these the most appropriate measures for answering the study question?
If the study compares different subgroups from the survey, were the data obtained using the same methods from these different groups?
How was the survey carried out?
Is the survey method likely to have introduced significant bias?
Have ethical issues been taken into account?
Is the study large enough?
Is there adequate description of the data?
Is there evidence of multiple statistical testing or large numbers of *post hoc *analyses?
Are the statistical analyses appropriate?
Is there evidence of any other bias?

## Results

The searches produced 346 papers; the abstracts of all papers identified were read in order to exclude those not meeting the inclusion criteria. However, the inclusion and exclusion criteria produced a collection of literature which was limited by the fact that there are few empirical studies on non-pharmacological forms of pain relief. Those excluded at this stage focused on the following: a specific type of pain relief (82), a measure of pain (37), another aspect of labour (120), a professional or personal viewpoint (30) and others (8). A total of 277 papers were excluded; 69 full articles were retrieved and subsequently, if included, appraised in full. Thirty-two papers met the inclusion criteria, 13 qualitative and 19 quantitative. Thirty-seven full text papers were excluded because the focus was on the experience of specific methods of pain relief (4), measurement of pain (4), attitudes and descriptions of labour and pain (13), midwives' perceptions (4), assessment of interventions (5), general satisfaction (5) or antenatal education (2). Uncertainty about inclusion was resolved by discussion between two reviewers (RT and JEL). Data were extracted from each paper using the appropriate appraisal tool (see Tables 1 and 2 and Additional files 1 and 2). The appraisal tools were used for extracting the details from the identified papers; they also provided a structured approach to assessing the quality of individual papers. Issues regarding quality, such as timing of questions or countries in which the study was undertaken, which may have an impact on interpretation, are referred to in the text.

Once all studies had been appraised, four key themes were identified: the level and type of pain, pain relief, involvement in decision-making and control. Within each theme the results were broken down into sections on expectations, experience and the gap between expectation and experience, in order to best address the research question. Tables detailing the studies are included in the results section along with a review of the quality of the paper according to the criteria set out in the methods.

### The level and type of pain

#### Expectations of the level and type of pain

Studies exploring the expectations of pregnant women about the level and type of pain vary in their results. Key issues identified in this literature include positive or negative perceptions of pain, the concept that pain in labour is different from pain in an illness and variation in the anticipated level of pain.

One large qualitative study in Australia described women's negative expectations of pain [25]. Women who were interviewed foresaw birth as a potentially negative experience that was shaped by their antenatal fear and concern about the anticipated severity of pain [25]. A study conducted in Jordan also found that 92% of the women in the study expected a negative experience of childbirth, either frightening (66%), very long (63%), too difficult (66%) or painful (78%) [26]. The findings that can be taken from this study are limited as both the cultural differences and differences in provision in maternity care between Jordan and western culture and medicine are great. In contrast, a Swedish study described women's positive expectations as linked to the perception of a positive outcome and found that although women found pain hard to describe and often did so in contradictory terms, "I think it's a happy pain, though its hell" (p. 107 of [27]), the transition for women as they became mothers gave pain a positive meaning [27]. However, this study was conducted postnatally in a birthing centre whose ethos was that of natural birth and pain bringing women closer to their babies; it is likely that this ethos, along with being questioned postnatally, influenced the positive expressions about pain. Waldenstrom and colleagues suggested that such positive attitudes to pain are probably an expression of satisfaction with coping with pain, rather than satisfaction with pain itself [28]. However, Salmon et al found that women's rating of the painfulness of labour were unrelated to feelings of achievement; in fact, a painful birth was just as likely to have a positive evaluation as a pain-free birth [29].

Two authors, in particular, argued that pain in labour is different from other pain and identified that there is a risk that we expect to treat pain in labour like an illness [27,30], that is, a side effect that needs to be eradicated. However, Green and colleagues found that not all women agreed with the concept that labour pain is different from the pain of an illness; it tended to be the better educated in their study that saw this difference [30].

The final issue relates to expectations of the severity of pain; several studies reported that women anticipated suffering extreme or unbearable pain during labour [31-33]. McCrea et al suggested that the women who expected labour to be "quite painful", on a five-point scale ranging from very painful to not at all painful, held realistic expectations of what labour would be like [33].

It is important to recognise the potential impact that these differences in expectations might have. As Fenwick and colleagues identified, choices that are made throughout labour are made on the basis of how women anticipate labour pain [25]. For example, if a woman views labour as a medical condition with risks, she may be more likely to choose pain relief to eradicate the pain. If, however, she views labour as a normal and natural process, she may be more likely to employ natural methods of coping and pain relief. One study found that expectations regarding the level of anticipated pain influenced a woman's perception or satisfaction with the birth experience, either negatively by feeling a failure as they were in greater pain than expected or positively by being pleasantly surprised as "torments which were expected" never came [34].

#### Experience of level and type of pain

The studies that focused on actual experience of pain in labour identified a wide range of experiences; one study found no difference in expectation and experience of pain levels [35]; in most studies [31,32,34,36-38] women found the pain worse than anticipated; in only one study did women report the pain to be better than expected [38]. The studies where the pain experienced was found to be worse than expected, in which women were questioned between 2 months and 20 years after birth, reported that this was especially true in the case of primiparous women [31,32,34,36-38]. Care does need to be taken when interpreting this data as recall may not be as accurate when talking about an event which happened 20 years ago. The one study that reported women's pain experience to be better, although different, than expected [38] found that three out of the eight women questioned described the labour overall as less painful but that the contractions were perceived as being more intense than expected [38]. Other unexpected qualities reported in this study related to the location of the pain rather than the severity, that is, pain in their back rather than in their abdomen, or in the pattern of pain, that is, pain coming in waves rather than being constant [38]. It is clear that the experience of pain for many women is different from anticipated. Following on from this, Waldenstrom et al stated that if women expect the worst pain imaginable then they will end up having a painful, negative experience, in contrast to women whose view was more optimistic, implying that your expectations shape your experiences [39].

#### Gap between expectation and experience of labour pain

Several studies identified a gap between expectation and reality [31,32,34,36-39] focusing particularly on the underestimation of pain.

The finding that women underestimate the level of pain is supported by several authors including Waldenstrom et al who specifically identified the underestimation of the 'intensity' as the primary reason for the gap in reality [40]. However, this was the only study where women were asked postnatally about their antenatal expectations and their actual experience; it may be difficult, after birth, to accurately recall antenatal expectations. In an antenatal questionnaire of 324 women, 36% anticipated suffering extreme pain, but 65% actually reported experiencing extreme pain before analgesia [31]. Once again differences between multiparous and primiparous women were found, with more primiparous women rating pain as worse than expected [39]. Green et al found that for 20% (*N *= 133) of women pain was not as expected, and for a further 38% (*N *= 252) it was as expected in some ways but not in others; the primary way it was different, reported by 20% (*N *= 143), was to be more painful [30].

The studies included largely show that women underestimate the intensity of the pain they will experience. If women are not able to have more accurate or realistic expectations about pain in labour they will not be able to prepare themselves appropriately for labour.

### Pain relief

#### Expectations of pain relief

Studies of women's expectations of pain relief found, unsurprisingly, that women wanted to access effective pain relief. A wide range of preferences was identified ranging from women wanting no drugs at all during labour to those requesting sufficient drugs to make it a manageable or pain-free experience.

The first of these issues was identified in a quantitative study where the authors concluded that modern pregnant women are well informed, expect to have effective pain relief and are disappointed if their wishes are not fulfilled. They argue that a woman needs to be prepared for the possibility of pain relief or she may feel disappointed, if she needs an epidural for example when she had not prepared for the possibility antenatally [41]. However, others have argued that by offering women this 'pain relief menu' we are undermining women and alternatively should be encouraging them to work with pain [42].

Several studies have commented on the level of pain relief women expected to achieve [32,38,43-45]. In a postal questionnaire survey, 67% of women wanted minimum drugs to keep the pain manageable, 22% said they would "put up with a lot of pain to have a drug free labour", whilst only 9% wanted the most pain-free labour drugs can give [32]. Rajan [44] and Ranta et al [45] identified women within their study groups who, when questioned antenatally, expected to be able to go through labour without any pain relief. Rajan identified 6% of the study population [44], whilst Ranta et al identified 4% of primiparous and 14% of multiparous women [45] who expected no pain relief during labour. In contrast, Beaton and Gupton demonstrated that women who had expressed a desire to avoid analgesia if possible also held realistic expectations by acknowledging that they would be willing to use drugs if necessary [43]. However, Gibbins and Thomson found that, although women were not sure what to expect from the pain during labour, they hoped it would be manageable, with or without analgesia [38].

#### Experience of pain relief

The literature on experience of pain relief methods focused on how expectations may or may not have an impact on experience, the numbers of people who actually had pain relief during labour, as well as people's knowledge and satisfaction regarding the experience of pain relief.

Two studies focused on how women's expectations concurred with their experiences. Fridh and Gaston-Johansson found that there was no significant difference between the medication women expected to use when questioned antenatally and the actual medication they used during labour [37]. In contrast, Green highlighted that the more painful women expected a drug-free labour to be, the more likely they were to actually use drugs, particularly in the case of pethidine [32].

An ethnographic study of 80 women looked at the expectations of women who had antenatal education from the National Childbirth Trust and other women who had not had any antenatal education. Although the National Childbirth Trust women expected a natural drug-free labour, there was no difference in the actual drugs administered between the groups during labour [46]. So although their expectations were different, their actual medication use was the same. The number of women who actually had some form of pain relief during labour varied between 84% and 100% [32,38,44,45]. In one study women felt that they had remained open minded and made the right decisions to use certain methods of pain relief at the right time [38]. As many as 97% in another study used some form of pain relief; the 3% who used no pain relief methods had not intended to do so originally [44]. Many used a combination of drugs, with gas and air (Entonox) being reported to being the most widely used, although some women saw it as "somehow 'natural' not really a drug at all" (p. 69 of [32]).

Capogna et al demonstrate that levels of knowledge of pain relief methods vary across Europe; for example, only 47% of Italians and 64% of Portuguese women were aware of epidurals, compared with 94–100% of British, Belgium and Finnish participants. It could be argued that this is more a reflection of the approach to availability and choice of pain relief in these countries rather than education [47].

Regardless of choice it is important that women are satisfied with the pain relief experienced. A study in Finland found the majority of women had a positive attitude to pharmacological pain relief postnatally, with 88% of the women having planned on requesting it [41].

#### Gap between expectation and experience of pain relief

An expectation-reality gap was identified where women expecting a drug-free labour did not have one. Of those women in Ranta et al's study who said they would not use pain relief, 52% actually used it [45], demonstrating a discrepancy between hopes and expectations and the actual experience of decisions or actions taken in labour. Although there was a gap identified between expectation and experience of pain relief, two studies made the distinction between the hopes of having a drug-free labour but the expectation that they may have to have some sort of pain relief [41], particularly if the labour was long [43].

### Involvement in decision-making

#### Expectations of involvement in decision-making

One of the questions that this systematic review aimed to answer was 'What is women's involvement in the decision-making process?'. What was found is that women are as concerned about being involved generally [33,48], that is, being in control [49] and being able to cope [38], as they are about being directly involved in the decision-making process. Whilst these are within the realm of decision-making, the women themselves rarely referred to decision-making explicitly. One study reported on what influenced women in their decision-making, stating that it was public discourses, for example, the media, rather than formal antenatal education that was most influential, with private discourses with friends and family also highly influential [25]. Lavender et al highlighted that 26% (108) of the women in their study acknowledged that they wanted to participate in decision-making, but the degree of involvement was different [50].

#### Experience of involvement in decision-making

The limited literature in this review on the experience of involvement in decision-making concentrated on the type of women who wanted to be involved and how antenatal education empowered women to become involved.

Firstly, according to McCrea et al, it is multiparous women who place emphasis on being fully informed rather than primiparous women who are concentrating on controlling emotions rather than being involved in decision-making [33]. Green and Baston support this, in that they found participation in decision-making was important to multiparous women, but being treated with respect and being treated as an individual was more important [51].

Secondly, regarding education, two studies reported that preparation helped women cope physically and psychologically with their labour; also their knowledge of pain relief helped them make informed choices [38,52]. However Carlton et al question whether some hospital-based education serves to socialise women about the "appropriate" ways of giving birth rather than educating them [52]. Brown and Lumley examined the use of birth plans and found that 21% (56 out of 270) of participants found them to be useful as it gave women an opportunity to consider and evaluate the options before labour began [53].

#### Gap between expectation and experience of involvement in decision-making

None of the studies included in this review reported a gap between expectations and experiences of being involved in decision-making. This is not to say that a gap does not exist, rather that such research has not been undertaken or published.

### Control

Green and Baston set out clear definitions for the different types of control, internal and external [51]; external control concerns what is done to you, often equated with involvement in decision-making, and internal control relates to control over the body or behaviour. With reference to a study by Walker et al, where the midwife took full control [54], Green had earlier questioned whether some women place greater weight on one form of control or another whilst others wish to be in control both internally and externally, to ensure a fulfilling labour [55].

#### Expectations of control

The studies which looked at expectations of control were limited but did differentiate between types of control. For example, in a study by Green and colleagues, 66% (*N *= 711) of women expected to be in control of staff, 37% (*N *= 397) expected to be in control of their own behaviour, that is, internal self-control, and 54% (*N *= 576) expected to be in control during contractions [32,51].

#### Experience of control

Literature examining women's experience of control looked at specific issues including control of their own behaviour, how pain was managed, what pain relief was administered and level of involvement.

Green and Baston examined control of staff, behaviour and contractions and found that only 21% of women (234) felt in control in all three areas and 20% (219) felt out of control for all three, whereas antenatally 66% (711) had expected to be in control of staff, 37% (397) in control of behaviour and 54% (576) in control of their contractions. Control of staff was related to interpersonal variables, for example, being supported led to increased levels of control; pain and methods of pain relief were the primary factors for feeling in control of behaviour, for example, low levels of pain were associated with increased feelings of control and use of Entonox was associated with a twofold decrease in control; finally, control of contractions was predicted primarily by the experience of pain and ability to get into the most comfortable positions [32,51].

One European study focused on the actual control of pain during labour; Capogna et al found that that those who anticipated being able to control pain were indeed able to control and bear more pain before they had any analgesia [49]. The literature on how pain was managed was supported by McCrea et al who reported that women felt that they were in control of how their labour pain was managed, rather than being in control of the actual pain. In this study, McCrea et al argue that control goes beyond decision-making and also includes women utilising personal coping strategies [33]. Given the importance of this sense of control, preparation of women for labour is crucial to allow them to take control and is, according to McCrea et al, not something that ought to be left to the last few weeks of pregnancy [33], as is the case with antenatal education in the UK which starts anywhere between 28 and 36 weeks. As demonstrated by McCrea et al [33], part of being in control of how labour is managed includes feeling in control of the pain relief being administered. Women are more likely to be satisfied if they are involved in decisions about the management of their labour, rather than if the decisions are taken out of their hands [33,56].

One study identified that a woman's choice of setting for birth may reflect the level of control she wants in labour at an early stage. In an American study, those who chose a community delivery articulated a need for a sense of control or the ability to meaningfully influence decisions, whereas the women who chose a hospital delivery emphasised the perceived safety of the medical model and focused on safe outcomes, rather than the desire for control and optimum birth experience [49]. As this was a study conducted in America, the culture of hospital births that is dominant within the American healthcare system should be noted. Machin and Scamell also commented that at a time of crisis the women in their study were reassured by the messages and equipment of medical staff [57]. The choice a woman makes regarding place of delivery has an impact not only on her approach to labour generally, but also on the pain relief options open to her as labour progresses.

#### Gap between expectation and experience of control

Davis-Floyd identified where expectations about control are poorly matched with experience [58]. She argues that this is not always a negative thing. In her study she found that even if the birth was not natural as planned, women were still pleased with the experience if they felt they had been in control of the decisions made [58]. This evidence lends support to the argument that it is important to clarify what are the most important issues to each woman during labour, that is, is it control or is it minimum pain or adequate pain relief? Clarification of what is important to each woman allows the midwife to fully support her throughout labour

## Discussion

This review has identified four major themes relevant to women's expectations about pain, its relief during labour and their involvement in the decision-making process, namely the level and type of pain, pain relief, involvement in decision-making and control. This has given insight into the areas of expectations and experience of pain and its relief in labour. The review has also shown that within each of the themes identified there is a mismatch or a gap between women's expectations and their experience.

A limitation of this review is that, owing to the relatively small number of studies, we had to include papers in which expectations about pain in labour were a secondary outcome. In some cases, because pain was not the primary focus of the research, detailed information was unavailable. Within this small number of studies the focus is on pharmacological forms of pain relief; a gap in the literature exists for evidence relating directly to non-pharmacological methods. A further limitation is that, although initially it was stated that we would investigate experience and expectations about decision-making in this area, the evidence in this area is weak, with it being at best a minor outcome of few studies.

The strength of this review is in providing an overview of the research in the field. It gives great insight into what women's expectations are, how their expectations match with their actual experience and what decisions are made.

The results of the studies included in this review have many implications for both practice and policy. To consider first the implication of how realistic expectations can be formed by pregnant women; Gibbens and Thomson found antenatal anxiety was associated with a less positive experience [38] and Green and Baston question whether an intervention to raise the expectations of pregnant women may result in better experiences [51]. If midwives were able to reduce the anxiety that women felt throughout pregnancy and equip them to form realistic expectations, they may be able to assist women in having a more positive experience. However, the importance of antenatal education remains high, with its potential to empower women with realistic expectations and to enable them to make informed decisions. What is not clear is whose responsibility it is to provide or seek the information, when is it most appropriate to give the information and to what format will the women be most receptive. A form of antenatal education needs to be delivered which gives expectant mothers a more realistic expectation of what is likely to happen in labour [37]. Without some form of education from health professionals, or childbirth educators, women have to rely on media, family and friends for information, which may not help in forming realistic expectations. Although not all women attend antenatal classes, it is a key vehicle for education and one which we can endeavour to change to provide a balanced approach to childbirth. It was identified that childbirth training and information on pharmacological pain relief should be regarded as compatible and complementary to other coping mechanisms. Women need to be prepared for the possibility of pain relief, otherwise feelings of disappointment may arise [41]. However, what is unclear from this body of literature is whose responsibility it is to ensure that women are fully prepared for labour. Are women who are expecting a drug-free labour being helped or hindered in forming realistic expectations about labour, as their expectations of a drug-free labour are often not met [30]? Antenatal preparation classes are seen as one a way of providing information to pregnant women, but it seems this is not enough to prepare women for the experience of labour; decision support or information is needed to fulfil women's needs. Waldenstrom et al found that those who had more severe pain had more often attended antenatal class [28], whilst Kangas-Saarela and Kangas-Kärki found that even though nine out of ten women attended antenatal class, fear of labour remained high [41]. This may imply that anxiety or issues of fear were not being addressed in the classes [46].

This review was unable to determine when and how decisions are made regarding pain relief in labour. If we are to provide decision support for women, then further research needs to be conducted to gain an insight into the decision-making process during pregnancy and labour. Much of the research in this review has pointed to the fact that professionals involved in the care of pregnant women help shape their expectations. However, further research needs to be undertaken to examine how best to support professionals to guide women to make decisions that are appropriate, realistic and satisfactory.

## Conclusion

If women are well prepared during pregnancy, then they are more likely to have realistic expectations of the levels of pain, less likely to feel a failure and have increased confidence, which in turn can lead to more a positive experience. Women may have ideal hopes of what they would like to happen, but they need to be educated or informed to ensure that they are prepared for what might actually happen and give them the tools to deal with this.

This review identified a gap, a mismatch between women's expectations and their actual experiences. There has been a mismatch between how painful women expect labour to be, how long it will last, what pain relief they will need, how in control they will be and what the actual experience is like. If we are to improve women's experience of labour, we need to look at how the expectations of these women can be brought more in line with their actual experience.

In conclusion, it may be that we now need to focus on a distinction that was made by Fenwick et al, Beaton and Gupton, and Gibbens and Thomson, among others, that women should have hopes of what they would like labour to be like, but should also have an understanding of what might happen. By distinguishing between the two, women can say what they would ideally like to happen, but also consider and recognise that things may not go according to plan and, if this is the case, be fully aware and prepared to make the necessary decisions.

## Competing interests

The author(s) declare that they have no competing interests.

## Authors' contributions

JEL, RGT, MJM and SM have all contributed to the development of the review. JEL identified and reviewed all papers and prepared the initial draft of this manuscript. RGT was the second reviewer. All authors reviewed the manuscript critically for content and approved the final version to be submitted

## Pre-publication history

The pre-publication history for this paper can be accessed here:



## Supplementary Material

Additional file 1 Qualitative papers included in review. Complete list of the qualitative papers included in this review. Click here for file

Additional file 2 Quantitative papers included in review. Complete list of the quantitative papers included in this review. Click here for file
