# Supplementary material for: More in hope than expectation: a systematic review of women's expectations and experience of pain relief in labour
Source: BMC Med. 2008 Mar 14;6:7. doi: 10.1186/1741-7015-6-7 (PMC2358911; doi:10.1186/1741-7015-6-7)
Supplement: Additional file 1 — Qualitative papers included in review. Complete list of the qualitative papers included in this review. [file 1741-7015-6-7-S1.doc]

| **Reference** | **Data Collection** | **Analysis** | **Key Findings** | **Appraisal** |
| --- | --- | --- | --- | --- |
| Beaton[1]  1990  Canada | In-depth Interviews in  In 3^rd^ trimester  N=11 | Content analysis | No basis for comparison of pain, largely the unknown that provoked fear and anxiety. Women often optimistic about ability to cope. Wanted to avoid analgesics if possible but willing if necessary.  Role of support: helping them maintain control and act as go-between with staff. | No mention of reflexivity  One of the childbirth educators recruited the women at the beginning of the class |
| Carlton[2]  2005  USA | In depth interviews  N=33 | Ethnographic | Whether women wanted a “safe” passage or an “enhanced” passage had strong influence on satisfaction. Midwives have the potential to help women have realistic expectations. Hospital based education helped to socialise women about ‘appropriate’ ways of giving birth rather than educating | Sample of participants contacted to reflect upon and clarify findings.  Not clear when interviews where conducted |
| Davis – Floyd[3]  1994  USA | In depth interviews  N=40 | Technocratic model | Most who gave birth in hospital felt pain was bad, not to feel pain was good and was their intrinsic right as a modern women.  Even if birth wasn’t natural as planned – still pleased if they felt in control of their decisions. The major discontents women expressed was not the administration of anaesthesia but withholding it | Little information on how the information was collected or analysed |
| Fenwick[4]  2004  Australia | Telephone interviews  with women who were pregnant or had given birth in last month  N=202 | Explorative descriptive design and constant comparison | Birth choices based on whether women expect labour to be a medical condition with risks or a normal natural process.  Often had expectations but also hopes of what would happen. | Description of data meetings and audit sample given of how the data was analysed and themes generated  Newspaper recruitment used to gain a wide range of women |
| Gibbens[5]  2001  England | Unstructured interviews at 38 weeks and 2 weeks Postnatally  N=8 | Phenomenological | Confidence links to higher expectations and positive experience. Anxiety associated with less positive expectations and experience. Midwives need to discover wishes of women to respect and support them. | Clear description of the analysis used.  Draft findings given to each participant for verification and anonymity. |
| Halldorsdottir[6]  1996  Iceland | Interactive interviews  N=14 | Phenomenological | Women’s circumstances affected their birth experience. Women felt a failure because they were in more pain than expected. They longed for a sense of security involving a need for information and explanation  If needs are fulfilled their experience is positive, midwives need to know what these needs are | Investigator triangulation, peer debriefing, member checks and others interviewed for referential adequacy. Reflexive journal kept |
| Hodnett[7]  2002 | Systematic Literature review | Pain and women’s satisfaction with the experience of childbirth | 4 factors identified – personal expectation, support from care-givers, quality of relationship with caregivers, involvement in decision making  Discrepancy theories predict satisfaction based on difference between what is expected and what is received. None of the studies distinguished between expectation and preferences. |  |
| Kabakian[8]  2000  Lebanon | Semi structured interviews within 3 months Postnatally  N=117 | Thematic | Belief from rural women that delivery entailed a lot of “suffering” for women and this was expected. Consequently many women did not request pain relief because they did not expect painless deliveries. In Beirut women demanded epidurals and strove for a painless delivery | No clear description of process of analysis or reflexivity of researchers.  Rural areas lack any infra-structure of health care provision at all – so limited generalisability |
| Lavender[9]  1999  England | Questionnaire 2 days Postnatally  N=412 | Thematic | Expectations played a part in whether they considered experience fulfilling. 34% felt unprepared for labour – either lack of information or unrealistic expectations. | Themes generated by 2 researchers independently |
| Lundgren & Dahlberg[10]  1998  Sweden | Interviews 2-4 days postnatally  N=4 primiparous , 5 multiparous woman | Phenomenological | Pain is hard to describe – it is contradictory.  Pain brought women closer to their baby. Risk that we compare and treat pain in childbirth as like pain in illness. Suggestion that ante-natal training involves intense training of the body | Bracketing of knowledge, experience of the phenomena |
| Lundgren and Dahlberg[11]  2004  Sweden | Synthesis of 4 studies  N=39 | Phenomenological | Women experience pain in a way that gave meaning to transition to motherhood. Control is important but means different things to different women. Midwives responsibility that women do not exceed the limit of their ability. | Not clear how the data were collected in the original studies.  Original interviews not read |
| Machin[12]  1998  England | Observations from early pregnancy to several months Postnatally  N=80 | Ethnographic | Information is always received by people who already have existing values and ideas. National Childbirth Trust women all felt empowered but didn’t go on to fulfil this in delivery. Cannot assume one message will be effective in a diverse society | No evidence of reflexivity – although data came from many different sources |
| Machin and Scamell[13]  1997  England | Interviews, participant and non-participant observation throughout pregnancy and childbirth  N=80 | Ethnographic | National Childbirth Trust were confident and wanted a natural childbirth. Women felt compelled to take responsibility. Those who received NHS antenatal care did not challenge interventionist medical approach, their priorities were only for a healthy baby and painless labour. Those receiving NHS antenatal care never aspired to notion of control. But as labour started women who had received National childbirth Trust antenatal education became vulnerable and accepting of the medical model | Discussion with researcher, re-assess priorities, check techniques |

1. Beaton J, Gupton A: **Childbirth expectations: a qualitative analysis**. *Midwifery* 1990, **6**:133-139.

2. Carlton T, Callister LC, Stoneman E: **Decision Making in Laboring Women: Ethical Issues for Perinatal Nurses**. *The Journal of Perinatal & Neonatal Nursing* 2005, **19**(2):145-154.

3. Davis-Floyd RE: **The technocratic body: American childbirth as cultural expression**. *Social Science & Medicine* 1994, **38**(8):1125-1140.

4. Fenwick J, Hauck Y, Downie J, Butt J: **The childbirth expectations of a self-selected cohort of Western Australian women**. *Midwifery* 2005, **21**(1):23-35.

5. Gibbins J, Thomson AM: **Women's expectations and experiences of childbirth**. *Midwifery* 2001, **17**(4):302-313.

6. Halldorsdottir S, Karlsdottir SI: **Journeying through labour and delivery: perceptions of women who have given birth**. *Midwifery* 1996, **12**(2):48-61.

7. Hodnett EDRNP: **Pain and women's satisfaction with the experience of childbirth: A systematic review**. *American Journal of Obstetrics & Gynecology* 2002, **186(5)**(Supplement):S160-S172.

8. Kabakian-Khasholian T, Campbell O, Shediac-Rizkallah M, Ghorayeb F: **Women's experiences of maternity care: satisfaction or passivity?** *Social Science & Medicine* 2000, **51**(1):103-113.

9. Lavender T, Walkinshaw SA, Walton I: **A prospective study of women's views of factors contributing to positive birth experience**. *Midwifery* 1999, **15**(1):40-46.

10. Lundgren I, Dahlberg K: **Women's experience of pain during childbirth**. *Midwifery* 1998, **14**(2):105-110.

11. Lundgren I: **Releasing and relieving encounters: experiences of pregnancy and childbirth**. *Scandinavian Journal of Caring Sciences* 2004, **18**(4):368-375.

12. Machin D, Scamell A: **Changing practice. Using ethnographic research to examine effects of 'informed choice'**. *British Journal of Midwifery* 1998, **6**(5):304-309.

13. Machin D, Scamell M: **The experience of labour: using ethnography to explore the irresistible nature of the bio-medical metaphor during labour**. *Midwifery* 1997, **13**(2):78-84.
