# Supplementary material for: More in hope than expectation: a systematic review of women's expectations and experience of pain relief in labour
Source: BMC Med. 2008 Mar 14;6:7. doi: 10.1186/1741-7015-6-7 (PMC2358911; doi:10.1186/1741-7015-6-7)
Supplement: Additional file 2 — Quantitative papers included in review. Complete list of the quantitative papers included in this review. [file 1741-7015-6-7-S2.doc]

| Study | Method | Main Outcome measures / Aim of study | Key Results | Appraisal |
| --- | --- | --- | --- | --- |
| Brown & Lumley[1] 1998  Australia | Population based postal survey.  6-7 months post-natally. N=1333. Response rate = 62.5% | Use of a written birth plan. Relationship between use and overall satisfaction and involvement in decision making | Women who wrote birth plan more likely to be happy with what was done to relieve pain - 49% vs. 28.5% and to report always having an active say in decisions - 46% vs 38.3% | Acknowledges under-representation of non-English speaking. Postal questionnaire– no obvious bias |
| Callister[2]  1995  USA | Phenomenological – mixed methods study.  N=60 | Utah test to measure belief and perceptions about childbirth including fear, locus of control, participation and personal values | Positive experience enhanced when congruency between the philosophy and expectations of childbearing women and their health care providers.  Women with Obstetrician emphasised the perceived safety of medical management and risk of childbearing. Community deliveries emphasised the need for control or ability to meaningfully influence decisions | No evidence or explanation of the analysis of the qualitative data |
| Capogna & Alahuhta.[3]  1996  Europe | Structured Interviews and Visual Analogue Scale satisfaction  Last months of pregnancy and 24 hours post-natally  N=611 (>100 women from 5 countries) | Expectations and experiences of pain and pain relief in primips | Maternal expectations differed between countries. Knowledge of pain relief varied between hospitals, 47% of Italians, 64% of Portuguese, 94-100% of British, Belgium and Finnish mothers were aware of epidural. Older mothers were best informed while younger mothers had more pain before analgesia.  Those who said they could control pain had more pain before analgesia. Mothers who expected more pain were more likely to be satisfied with analgesia p=<0.001.Maternal satisfaction positively correlated with pain expected (SR.0.25 p=<0.001). | Birth itself approached differently in some countries  Timing of the post-natal interview could introduce bias |
| Fridh & Gaston-Johansson. [4]  1990  Sweden | Expectation questionnaire, midwifery assessment and Visual Analogue Scale.  32 weeks ante-natally, during labour and 2 days post-natally  N=50 primiparous and 88 multiparous | Assessment of expectation, in-labour pain and relationship between expected and actual pain | Primiparous women rated actual pain worse than expected.  No significant difference between expected and actual medication needed.  Neither group able to predict degree of pain | No mention of non-responders.  Not clear if it was a different midwife who scanned women to the one who recruited |
| Green, Kitzinger, Coupland.[5]  1990  England | Postal questionnaire  30-32, 36 weeks ante-natally and 6 weeks post-natally  N=751  Response rate = 74%, 92% and 96% | Attitudes, knowledge and expectations  Edinburgh Post Natal Depression scale | The more years of education the more importance placed on being informed. Women educated beyond 16 more likely to expect some control over non-emergency decision making. 2/3rds of women who had ceased education at 16 wanted to at least have decisions discussed.  20% pain in labour was not as expected | limited results, repeated in greater detail in a later paper |
| Green [6]  1993  England | Postal Questionnaire.  28-30, 36 weeks ante-natally and 6 weeks post-natally  N=751  Response rate = 74%, 92% and 96% | Overall satisfaction and wellbeing.  Edinburgh Post Natal Depression scale | 77% expect pain to be quite or very painful.  Strong relationship between worried about labour pain and worried about pain in ordinary life  20% found pain not as expected  Strong relationship between expectation and experience. | Postal – no response bias noted.  Asked questions about drugs to a further 853 women to validate the response |
| Green & Baston[7]  2003  England | Postal Questionnaire  29 and 35 weeks antenatally and 6 weeks postnatally (based on repeated “Great expectations” study by Green 1990)  N=1146  Response rate = 59.9%, 90.86% and 92.19% | Assessment of control outcomes: control of what staff do, control of own behaviour and control during contractions | 20% felt in control in all three ways, 20% felt out of control for all three.  67% expected to be in control of staff, 37% in control of behaviour and 53.8% in control during contractions.  For multiparous women expectations of control of behaviour and during contractions were major predictors of their experience | Replication of “Great expectations” study  Largely representative sample although biased towards well educated and those whose partners were employed |
| Kangras-Saarela & Kangas-Karki[8]  1994  Finland | Questionnaire, 1 day Postnatally  N=339  Response rate = 82% | Evaluate attitude to labour and pain relief | Majority had positive attitude to pain relief – 88% had planned to request it  In 50% of cases the midwife suggested pain relief. 43% pleased with pain relief, 17% disappointed. | Limited satisfaction scoring – very pleased/fairly/disappointed |
| McCrea, Wright & Stringer[9]  2000  Ireland | Rules questionnaire within 48 hours of delivery  N=300 | Assess rule governed behaviours relating to control of pain management in labour. | Primiparous women concerned ante-natally with controlling emotions. Multiparous women placed more emphasis on being fully informed. Little difference between those rules before childbirth and those that applied in practice. | Observational data reflected what women said.  Excluded women who had epidural as not involved in decision about pain relief |
| McCrea, Holly Wright[10]  1999  Ireland | Questionnaire, within 48 hours of delivery  N=100 | Examine influence of personal control on women’s satisfaction with pain relief | Expected labour to be “quite painful” and were “worried about pain”  Personal control a fact which could influence satisfaction. | Bias discussed – however, asking within 48hours – may skew results |
| Oweis  2004  Jordan | Questionnaire during pregnancy.  N=77 | Expectation of childbirth experience, nursing and support during labour and birth | Most women expected a negative childbirth experience.  92% scored >75 – average 85 out of 100.  78% expected it to be painful | No ante-natal education provided. No family member allowed in to support during birth – limited generalisability |
| Peach [11]  1991  Australia | Survey, within 24hours of delivery  N=1000 | Pain experience, analgesia and satisfaction. | More pain than expected reported by Primiparous women . Dissatisfaction more likely to be associated with instrumental delivery rather than pain. Satisfaction with medical care determined by explanations and participation in decision making. | Results given in numbers rather than % - misleading as very small percentages |
| Rajan[12]  1993  England | Secondary analysis of National Birth Survey.  During labour and within 6 weeks post-natally  N= 6459 and 1149 | Perceptions of women’s experience of pain and pain relief and the professionals who attended them. | 6% planned on not using pain relief, only 3% actually did not. Non-pharmacological methods tended not to be included in the methods the clinicians talked about. Clinicians tended to give primary consideration to the method that fell into their remit to administer. | Limitation of secondary analysis of data – leaves gaps that are unanswered |
| Ranta[13]  1995  Finland | Ante-natal survey  N=360 primiparous / 731 multiparous  Response rate= 86% | Expectations of level of pain and anticipated need for pain relief. | 96% received sufficient information.  4% multiparous and 14% primiparous (p<0.0005) expected not to use analgesia. 20% actually received no pain relief.  95% satisfied with care despite difference in pain relief management.  Those having instrumental deliveries most dissatisfied | Pain intensity recorded by midwife -  Subjective measurement |
| Salmon et al[14]  1990  England | Antenatal and Postnatal questionnaire  N=106 and 82  Response rate = 92% and 95% | Identification and rating of the dimensions women use to evaluate their experiences. | Evaluation of childbirth multi-dimensional. Women’s ratings of the painfulness of childbirth unrelated to either their feelings of achievement or the extent to which they described their experience as pleasant or unpleasant. A painful birth is just as likely to have positive evaluation as a pain free one. | Actual methods very vague – no details of actually how it was done and how the ratings were made and at what time points ante and post-natally |
| Shapiro et al[15]  1997  Israel | Structured interview one day Postnatally  N=324 | Maternal perception of delivery room experience | Significant difference of pain relief administered to primiparous and multiparous (p<0.0001).  45% of primiparous and 36% of multiparous women anticipated suffering extreme or unbearable pain.  Before analgesia 68% of primips and 65% of multips described pain as unbearable | Unclear how sample reflects population. |
| Slade el al[16]  1993  England | Questionnaire at 36-39 weeks and within 72 hrs Postnatally  N=81 | Emotional, medical and control aspects of labour. | Expectations and experience of pain were similar. Duration of labour was sig. underestimated.  Women who expected to have medication or epidural generally did.  Expectations regarding pain levels not sig. different from those experienced.  Expectations and experience of pain did not affect satisfaction. | Conducted in ante-natal clinics and on post natal wards – could have affected the responses given. |
| Waldenstrom[17]  1996  Sweden | Questionnaire within 45 hrs after birth.  N=295  Response rate= 88% | Experience of pain intensity and affective dimension in relation to expectation. | 52% experienced pain as more difficult that expected.  Women with severe pain had more often attended ante-natal class, expected labour to be painful, dissatisfied with info and support and received more pain relief.  40% described pain as negative, 28% as positive. | Retrospectively asked them about expectations and experiences. The birth experience may colour what they remember their expectations being |
| Wight et al[18]  2000  Ireland | Questionnaire prior to and 72 hours Postnatally  N= 50 primiparous and 50 multiparous women | Rules held prior to and after childbirth | Little difference in rules held by women and midwives. Findings showed women’s expectations met with their experiences. Multips now feel they know how much pain they can bear and should therefore make decisions | No details on how the study was conducted  Observation conducted to record what actually happened to see if this matched up with what the women recorded happened. |

1. Brown SJ, Lumley J: **Communication and decision-making in labour: do birth plans make a difference?** *Health Expectations* 1998, **1**(2):106-116.

2. Callister L: **Beliefs and perceptions of childbearing women choosing different primary health care providers**. *Clin Nurs Res* 1995, **4**(2):168-180. (144 ref).

3. Capogna G, Alahuhta S, Celleno D, De Vlieger H, Moreira J, Morgan B, Moore C, Pasqualetti P, Soetenst M, Van Zundertl A *et al*: **Maternal expectations and experiences of labour pain and analgesia: a multicentre study of nulliparous women**. *International Journal of Obstetric Anesthesia* 1996, **5**(4):229-235.

4. Fridh GG-J, F.: **Do primiparas and multiparas have realistic expectations of labour**. *Acta obstetricia et Gynecologica Scandinavica* 1990, **69**:103-109.

5. Green JM, Kitzinger JV, Coupland VA: **Stereotypes of childbearing women: a look at some evidence**. *Midwifery* 1990, **6**(3):125-132.

6. Green JM: **Expectations and experiences of pain in labour: Findings from a large prospective study**. *Birth* 1993, **20**(2):65-72.

7. Green JM, Baston HA: **Feeling in control during labor: concepts, correlates, and consequences**. *Birth* 2003, **30**(4):235-247.

8. Kangas-Saarela T, Kangas-Kärki K: **Pain and pain relief in labour: parturients' experiences**. *International Journal of Obstetric Anaesthesia* 1994, **3**:67-74.

9. McCrea H, Wright M, Stringer M: **The development of a scale to assess control in pain management during labour**. *Journal of Reproductive and Infant Psychology* 2000, **18**:105-115.

10. McCrea B, Wright M, Holly E: **Satisfaction in childbirth and perceptions of personal control in pain relief during labour**. *J Adv Nurs* 1999, **29**(4):877-884.

11. Peach MJ: **The King Edward Memorial Hospital 1000 Mother Survey of Methods of Pain Relief in Labour**. *Anaesthesia & Intensive Care* 1991, **19**(3):393-399.

12. Rajan L: **Perceptions of pain and pain relief in labour: the gulf between experience and observation**. *Midwifery* 1993, **9**(3):136-145.

13. Ranta P, Spalding M, Kangas-Saarela T, Jokela R, Hollmen A, Jouppila P, Jouppila R: **Maternal expectations and experiences of labour pain--options of 1091 Finnish parturients**. *Acta Anaesthesiologica Scandinavica* 1995, **39**(1):60-66.

14. Salmon P, Miller, R.,Drew, N.C.: **Women's anticipation and experience of childbirth: The independence of fulfilment, unpleasantness and pain**. *British Journal of Medical Psychology* 1990, **63**:255-259.

15. Shapiro A, Fredman B, Zohar E, Olsfanger D, Jedeikin R: **Delivery room analgesia: an analysis of maternal satisfaction**. *International Journal of Obstetric Anesthesia* 1998, **7**(4):226-230.

16. Slade P, MacPherson SA, Hume A, Maresh M: **Expectations, experiences and satisfaction with labour**. *British Journal of Clinical Psychology* 1993, **32**(Pt 4):469-483.

17. Waldenstrom U, Borg IM, Olsson B, Skold M: **The childbirth experience: a study of 295 new mothers**. *Birth: Issues in Perinatal Care* 1996, **23**(3):144-153.

18. Wright ME, McCrea H, Stringer M, Murphy-Black T: **Personal control in pain relief during labour**. *J Adv Nurs* 2000, **32**(5):1168-1177.
